# Supplementary material for: One hundred years of zoonoses research in the Horn of Africa: A scoping review
Source: PLoS Negl Trop Dis. 2021 Jul 16;15(7):e0009607. doi: 10.1371/journal.pntd.0009607 (PMC8318308; doi:10.1371/journal.pntd.0009607)
Supplement: S1 Table — (DOCX) [file pntd.0009607.s001.docx]

**S1 Table. Preferred Reporting Items for Systematic reviews and Meta-Analyses extension for Scoping Reviews (PRISMA-ScR) Checklist (adapted from Tricco *et al.* 2018)**

| **SECTION** | **ITEM** | **PRISMA-ScR CHECKLIST ITEM** | **REPORTED ON PAGE #** |
| --- | --- | --- | --- |
| **TITLE** | | | |
| Title | 1 | Identify the report as a scoping review. | See Title |
|  |  | “ One hundred years of zoonoses research in the Horn of Africa: a scoping review” |  |
| **ABSTRACT** | | | |
| Structured summary | 2 | Provide a structured summary that includes (as applicable): background, objectives, eligibility criteria, sources of evidence, charting methods, results, and conclusions that relate to the review questions and objectives. | See abstract |
| **INTRODUCTION** | | | |
| Rationale | 3 | Describe the rationale for the review in the context of what is already known. Explain why the review questions/objectives lend themselves to a scoping review approach. | See Introduction |
| Objectives | 4 | Provide an explicit statement of the questions and objectives being addressed with reference to their key elements (e.g., population or participants, concepts, and context) or other relevant key elements used to conceptualize the review questions and/or objectives. | See introduction |
|  |  | This review aimed to address the following research questions:   1. What is the geographic focus of zoonotic disease research in the Horn of Africa? 2. What specific zoonotic diseases have been prioritized for research in the Horn of Africa? How does this align with country priorities? 3. What data are reported (human/animal/environment) in zoonotic disease research in the Horn of Africa? To what extent does zoonotic disease research meet the criteria for One Health research as reported in COHERE standards? 4. What methods have been adopted for zoonotic disease research in the Horn of Africa? 5. Which countries are author(s) affiliated to for zoonotic disease research in the Horn of Africa? For multi-author papers, to what extent is south-south and north-south collaboration occurring? |  |
| **METHODS** | | | |
| Protocol and registration | 5 | Indicate whether a review protocol exists; state if and where it can be accessed (e.g., a Web address); and if available, provide registration information, including the registration number. | N/A |
| Eligibility criteria | 6 | Specify characteristics of the sources of evidence used as eligibility criteria (e.g., years considered, language, and publication status), and provide a rationale. | See Methods, search strategy section |
|  |  | Database searches were performed within a day, on September 21st, 2018. Papers were included if they were in English or French, and published between 1918 and 2018 (i.e. 100 years). |  |
| Information sources | 7 | Describe all information sources in the search (e.g., databases with dates of coverage and contact with authors to identify additional sources), as well as the date the most recent search was executed. | See Methods, search strategy section |
|  |  | PubMED, Web of Science, Scopus, Cab Direct and ProQuest Dissertations and Theses. |  |
| Search | 8 | Present the full electronic search strategy for at least 1 database, including any limits used, such that it could be repeated. | See Methods, Table S4 and limitations in the discussion |
| Selection of sources of evidence | 9 | State the process for selecting sources of evidence (i.e., screening and eligibility) included in the scoping review. | See methods, screening and data extraction section |
| Data charting process | 10 | Describe the methods of charting data from the included sources of evidence (e.g., calibrated forms or forms that have been tested by the team before their use, and whether data charting was done independently or in duplicate) and any processes for obtaining and confirming data from investigators. | See Methods, data analysis section |
| Data items | 11 | List and define all variables for which data were sought and any assumptions and simplifications made. | See Methods, screening and data extraction sections, S3, S5 and S6 Tables |
|  |  | Data extracted on country, disease, domain and method |  |
| Critical appraisal of individual sources of evidence§ | 12 | If done, provide a rationale for conducting a critical appraisal of included sources of evidence; describe the methods used and how this information was used in any data synthesis (if appropriate). | N/A |
| Synthesis of results | 13 | Describe the methods of handling and summarizing the data that were charted. | See Methods, data analysis section |
| **RESULTS** | | | |
| Selection of sources of evidence | 14 | Give numbers of sources of evidence screened, assessed for eligibility, and included in the review, with reasons for exclusions at each stage, ideally using a flow diagram. | See Fig 1 |
| Characteristics of sources of evidence | 15 | For each source of evidence, present characteristics for which data were charted and provide the citations. | See Fig. 1 |
|  |  | All online sources were merged for analysis |  |
| Critical appraisal within sources of evidence | 16 | If done, present data on critical appraisal of included sources of evidence (see item 12). | N/A |
| Results of individual sources of evidence | 17 | For each included source of evidence, present the relevant data that were charted that relate to the review questions and objectives. | See Figures 2-5 and Tables 1-4 |
| Synthesis of results | 18 | Summarize and/or present the charting results as they relate to the review questions and objectives. | See Figures 2-4 and Tables 1-4 |
| **DISCUSSION** | | | |
| Summary of evidence | 19 | Summarize the main results (including an overview of concepts, themes, and types of evidence available), link to the review questions and objectives, and consider the relevance to key groups. | See Discussion |
| Limitations | 20 | Discuss the limitations of the scoping review process. | See Discussion |
| Conclusions | 21 | Provide a general interpretation of the results with respect to the review questions and objectives, as well as potential implications and/or next steps. | See Discussion and conclusion |
| **FUNDING** | | | |
| Funding | 22 | Describe sources of funding for the included sources of evidence, as well as sources of funding for the scoping review. Describe the role of the funders of the scoping review. | See funding section |

*From:* Tricco AC, Lillie E, Zarin W, O'Brien KK, Colquhoun H, Levac D, et al. PRISMA Extension for Scoping Reviews (PRISMAScR): Checklist and Explanation. Ann Intern Med. 2018;169:467–473. [doi: 10.7326/M18-0850](http://annals.org/aim/fullarticle/2700389/prisma-extension-scoping-reviews-prisma-scr-checklist-explanation)
