# Supplementary material for: One hundred years of zoonoses research in the Horn of Africa: A scoping review
Source: PLoS Negl Trop Dis. 2021 Jul 16;15(7):e0009607. doi: 10.1371/journal.pntd.0009607 (PMC8318308; doi:10.1371/journal.pntd.0009607)
Supplement: S2 Table — (DOCX) [file pntd.0009607.s002.docx]

**S2 Table. Final search terms.** For the disease specific search strategy, search terms were combined as follows: A1 **AND** (B1 **OR** B2 **OR** B3 … **OR** B14). For the general search, search terms were combined as follows: A1 **AND** C1 **NOT** (B1 **OR** B2 **OR** B3 … **OR** B14). Minor adaptations were made when conducting searches in each database to ensure similar types of search results.^a,b,c,d,e^ In particular, these adaptations were needed to avoid spurious search results based on the algorithm used by each database, e.g. when country or disease terms appeared only in the author affiliation, or when disease terms mapped to a larger concept like “parasite”.

| **Search terms/strategy** | | **Search string** |
| --- | --- | --- |
| ***A. Country search terms*** | |  |
| A1 | Countries/region of interest | Ethiopia OR Eritrea OR Somalia OR Somaliland OR Djibouti OR Kenya OR Sudan OR "South Sudan" OR Uganda OR "Horn of Africa" |
| ***B. Disease-specific search*** | |  |
| B1 | Gastrointestinal (zoonotic) | “non-typhoidal salmonella” OR “nontyphoidal salmonella” OR ((shiga OR shigatoxin OR enteropathogenic OR enterotoxigenic OR enteroaggregative) AND "Escherichia coli”) OR listeria OR campylobacter |
| B2 | Leptospirosis | leptospirosis OR “Weil disease” OR "Weil's disease" OR “canicola fever” OR “hemorrhagic jaundice” OR “mud fever” OR “swineherd's disease” OR leptospira |
| B3 | Cysticercosis | cysticercosis OR "pork tapeworm" OR “Taenia solium” |
| B4 | Tuberculosis (zoonotic) | “bovine tuberculosis” OR “mycobacterium bovis” |
| B5 | Rabies | rabies |
| B6 | Leishmaniasis | ((leishmaniasis OR “kala azar” OR “kalaazar” OR “kala-azar”) AND (zoonosis OR zoonoses OR zoonotic)) |
| B7 | Brucellosis | brucellosis OR “undulant fever” OR “malta fever” OR “Mediterranean fever” OR brucella |
| B8 | Echinococcosis | echinococcosis OR hydatid OR “echinococcus granulosus” |
| B9 | Toxoplasmosis | toxoplasmosis OR toxoplasma |
| B10 | Q fever | “Q fever” OR “query disease” OR coxiellosis OR “coxiella burnetii” |
| B11 | Trypanosomiasis (zoonotic) | ((trypanosomiasis OR trypanosomosis OR "sleeping sickness") AND (zoonosis OR zoonoses OR zoonotic)) OR “Trypanosoma brucei rhodesiense” |
| B12 | Anthrax | anthrax OR “malignant pustule” OR “malignant edema” OR “woolsorters disease” OR "woolsorter's disease" OR “Bacillus anthracis” |
| B13 | Hepatitis E | “Hepatitis E” |
| B14 | Rift Valley fever | “Rift Valley fever” |
| ***C. General zoonoses search*** | |  |
| C1 | General zoonoses | zoonosis OR zoonoses OR zoonotic |

^a^ For PubMed: search was limited to documents which included country search terms in title/abstract/MeSH terms only.

^b^ For Scopus: search was limited to journal articles which included country and disease terms in article title, abstract and keywords only.

^c^ For Web of Science: search was limited to documents which included country and disease terms in the “topic” (i.e. title, abstract, keywords) and was further refined by excluding the following document types: biography, data set, correction, meeting, news, abstract, book and patent.

^d^ For CAB Abstracts: search was limited to documents which included country terms in the title, abstract or geographic location field only.

^e^ For ProQuest theses: search was limited to documents which included country search terms in title or abstract.
