# Supplementary material for: One hundred years of zoonoses research in the Horn of Africa: A scoping review
Source: PLoS Negl Trop Dis. 2021 Jul 16;15(7):e0009607. doi: 10.1371/journal.pntd.0009607 (PMC8318308; doi:10.1371/journal.pntd.0009607)
Supplement: S3 Table — (DOCX) [file pntd.0009607.s003.docx]

**S3 Table. Criteria used to define which One Health domains are covered by a publication.**

| Data reported: | | | | |
| --- | --- | --- | --- | --- |
| Human domain | Animal domain | Animal product | | Environment domain |
| Collection and/or analysis of biological samples (e.g. faeces, blood, tissues) from humans  **OR**  Analysis of isolates from humans  **OR**  Survey or other data collection and/or analysis on knowledge, attitudes or practices that *influence human risk**  *Where exposure to animals/animal products/environment were considered as risk factors for humans, studies were classified as human domain only, unless criteria for other domains were also met. In this case, multiple domains were selected | Collection and/or analysis of biological samples (e.g. faeces, blood, tissues, milk, ectoparasites) from animals (mammals and birds) on farm or at slaughter  **OR**  Analysis of isolates from animals  **OR**  Survey or other data collection and/or analysis of practices or factors that *influence animal risk* | | Collection and/or analysis of animal products at harvest (milk, eggs) or post-harvest (meat, milk, eggs)  **OR**  Analysis of isolates from animal products  **OR**  Survey or other data collection and/or analysis related to value chain analysis of animal products | Collection and/or analysis of data from:   - biotic source (e.g. free-living arthropods, plants, plant-based food, manure) - abiotic source (e.g. water/soil/surfaces)   **OR**  Analysis of isolates/extracts from biotic/abiotic sources  **OR**  Survey or other data collection and/or analysis taking into account biotic/abiotic features that *influence risk to humans and/or animals* |
|  | - Studies involving sampling of milk from cows or eggs from poultry on farms (i.e. at harvest) was classified as both animal and animal product - Sampling of carcasses and offal in abattoirs (i.e. at harvest) was classified as animal domain only - Sampling of retail meat/milk/eggs (i.e. post-harvest) was classified as animal product only | | |  |
