# Supplementary material for: One hundred years of zoonoses research in the Horn of Africa: A scoping review
Source: PLoS Negl Trop Dis. 2021 Jul 16;15(7):e0009607. doi: 10.1371/journal.pntd.0009607 (PMC8318308; doi:10.1371/journal.pntd.0009607)

**S1 Fig. Number of publications on zoonoses, by year and disease, across all countries of the Horn of Africa (A) and in Ethiopia (B), Kenya (C), Uganda (D), Sudan/South Sudan (E), Somalia (F), Djibouti (G), Eritrea (H) and those with a regional focus (I).** Note: The bars in red represent the year the concerned zoonoses were prioritized in respective national workshops. In cases where no publications were produced that year, the year is indicated by a star. A solid light grey plot means there is no publication for a given disease in this country.

(A)
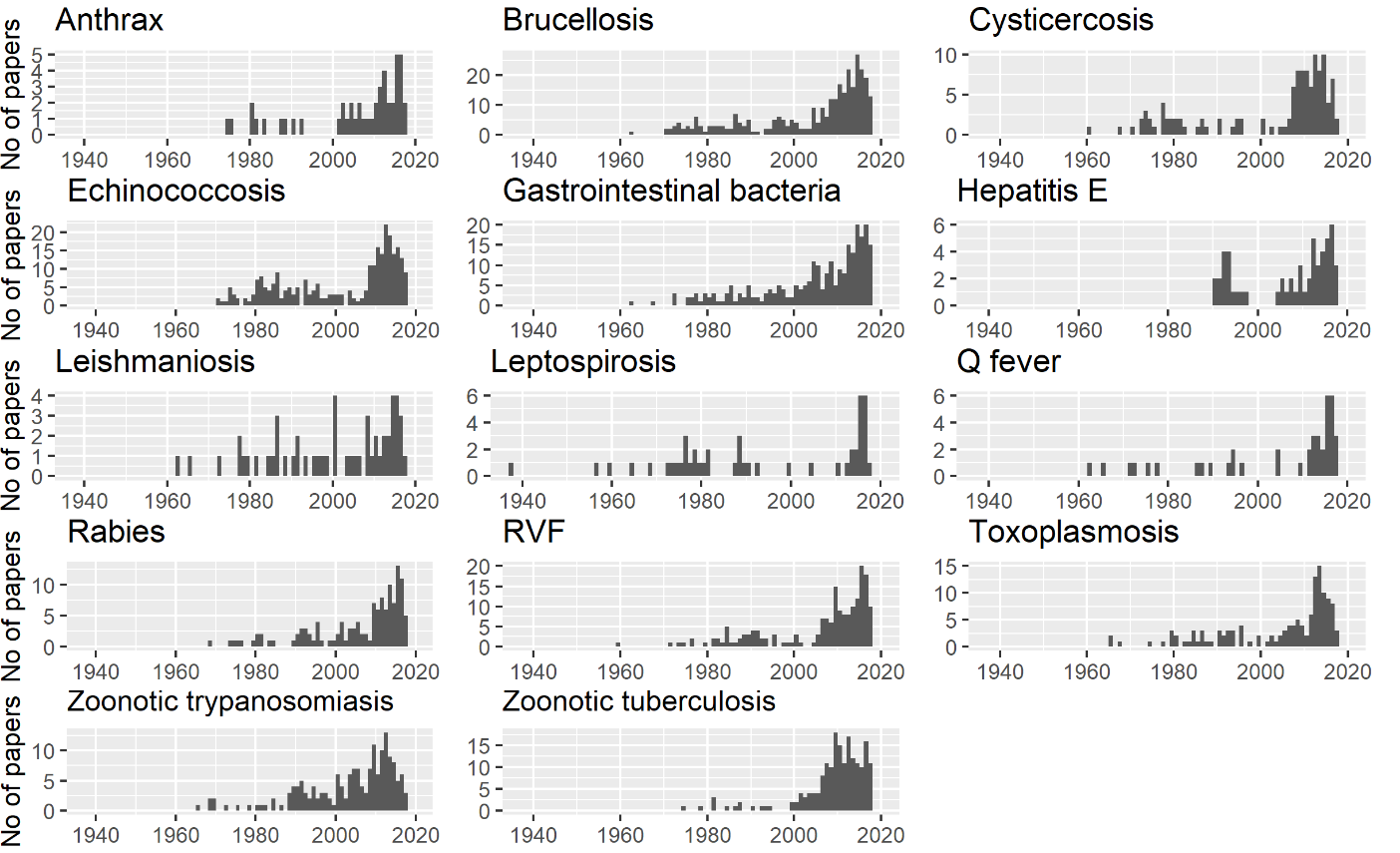


(B)
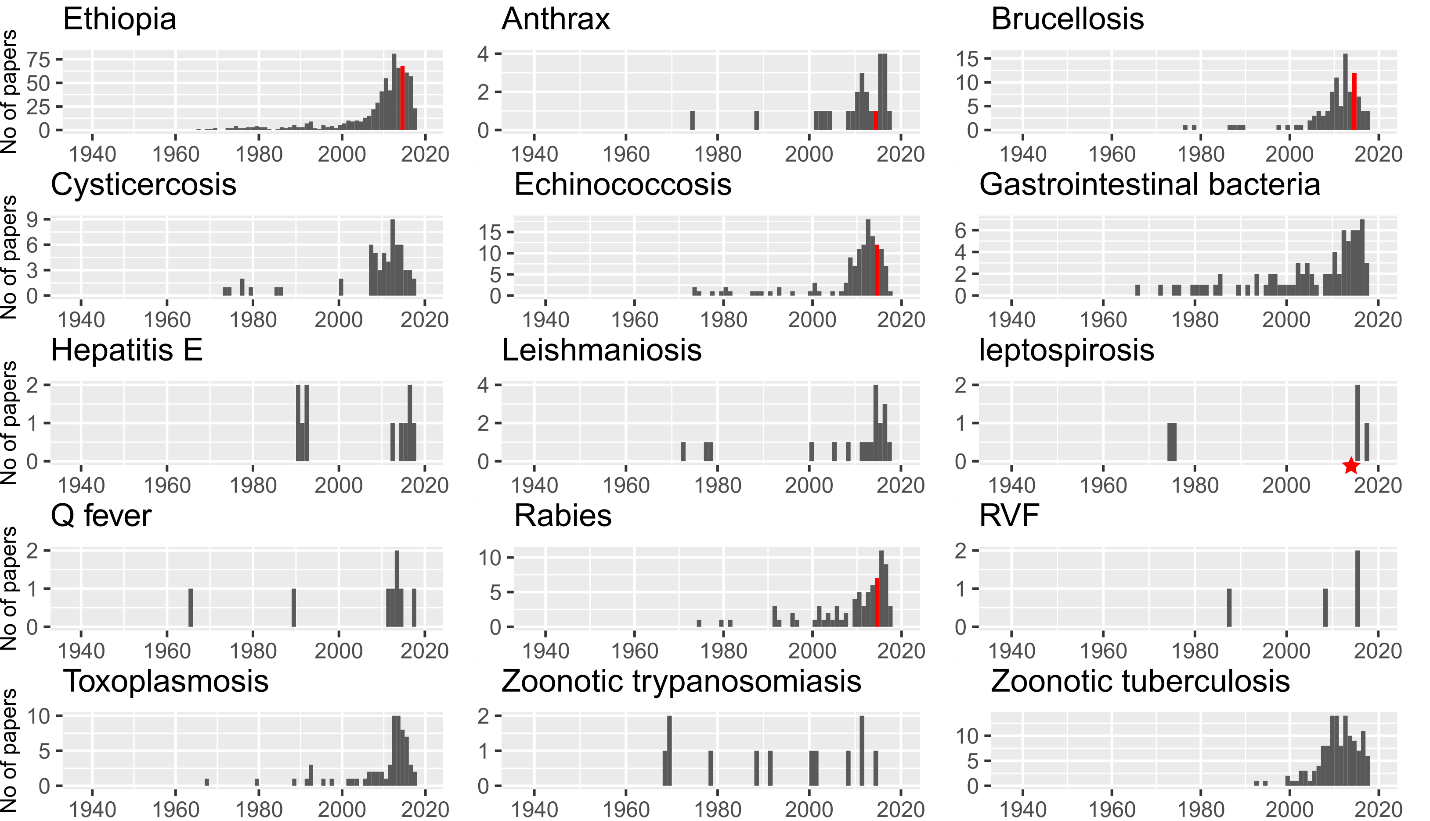


(C)


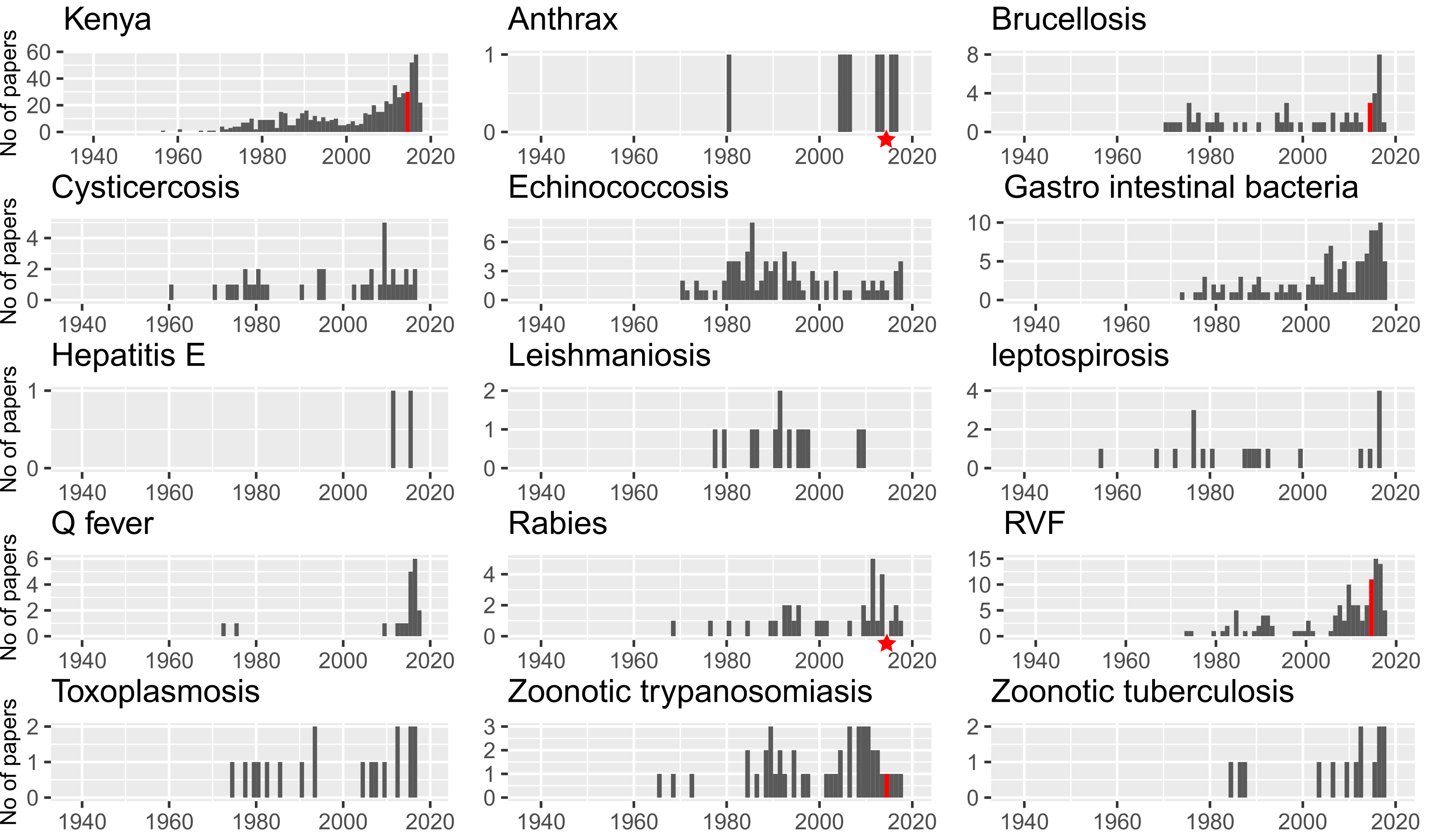


(D)
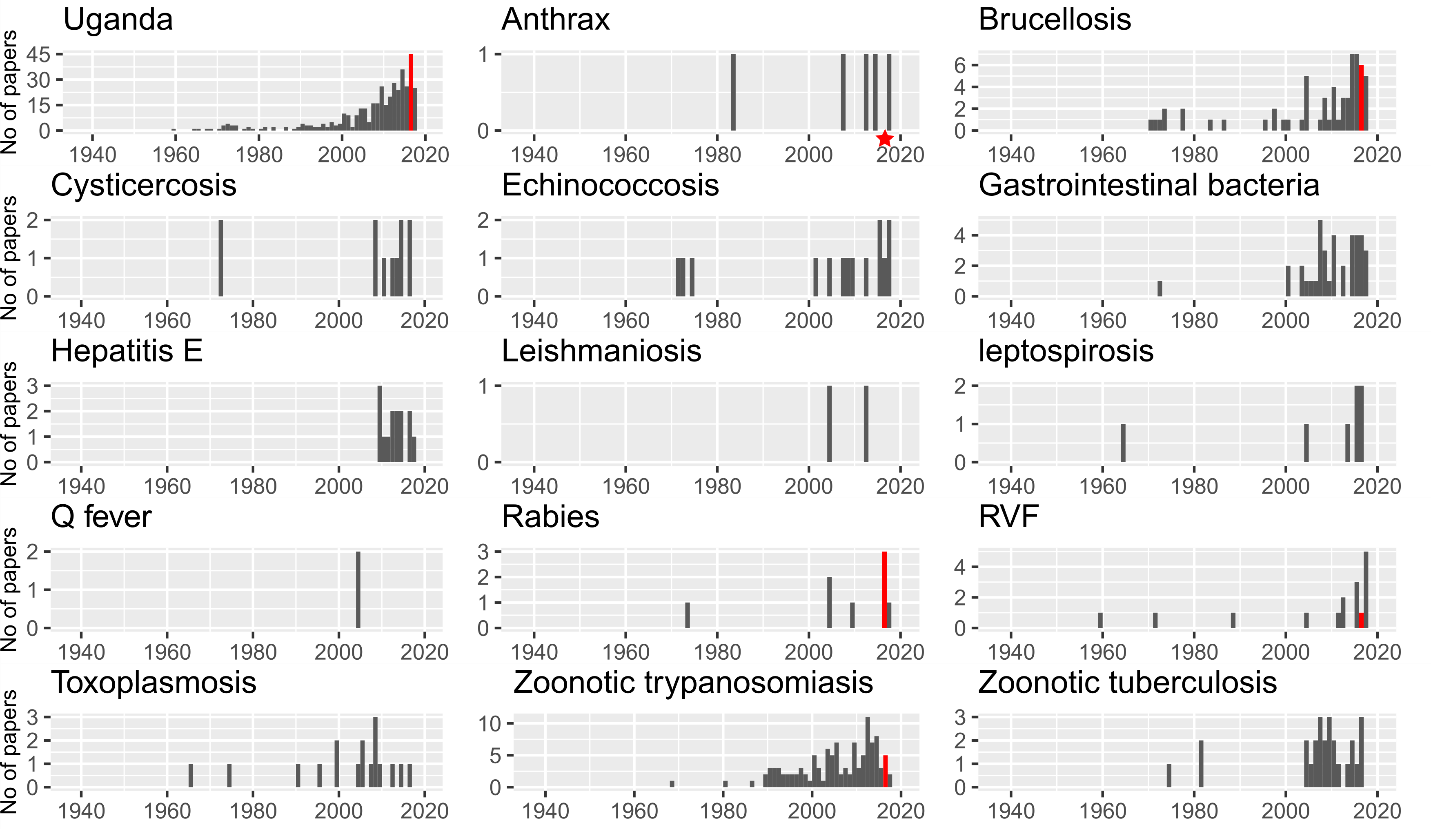


(E)


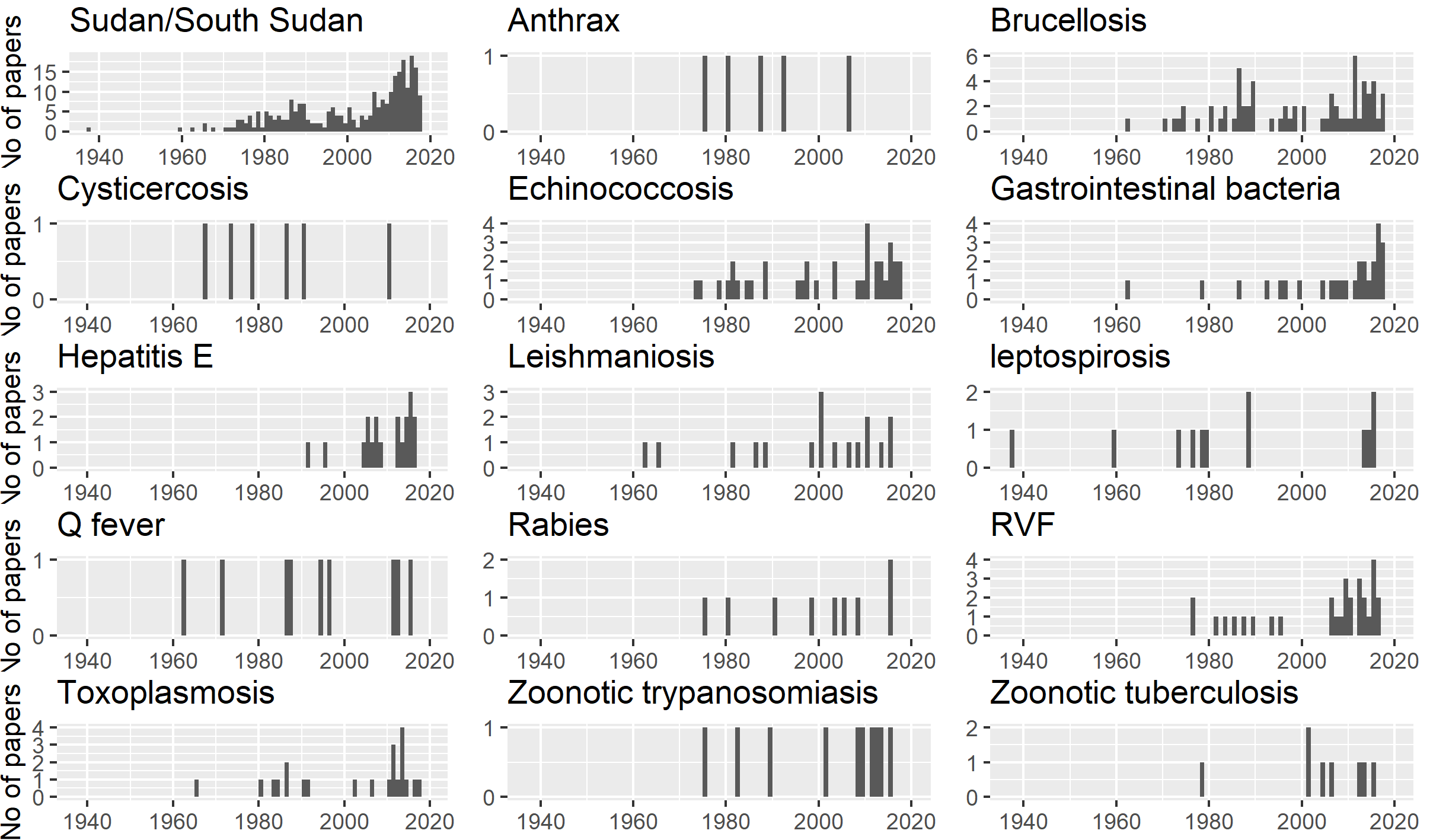


(F)


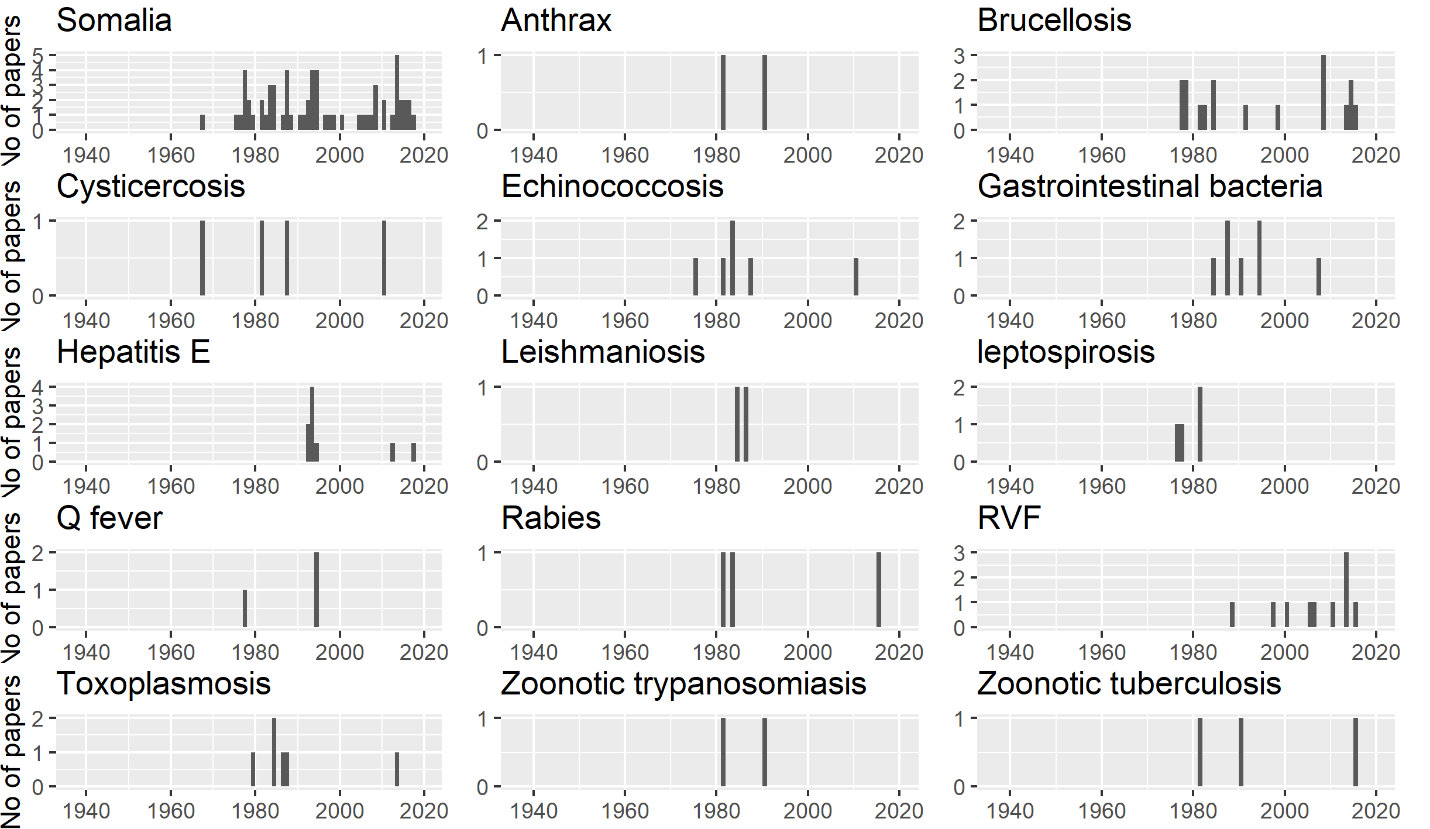


(G)


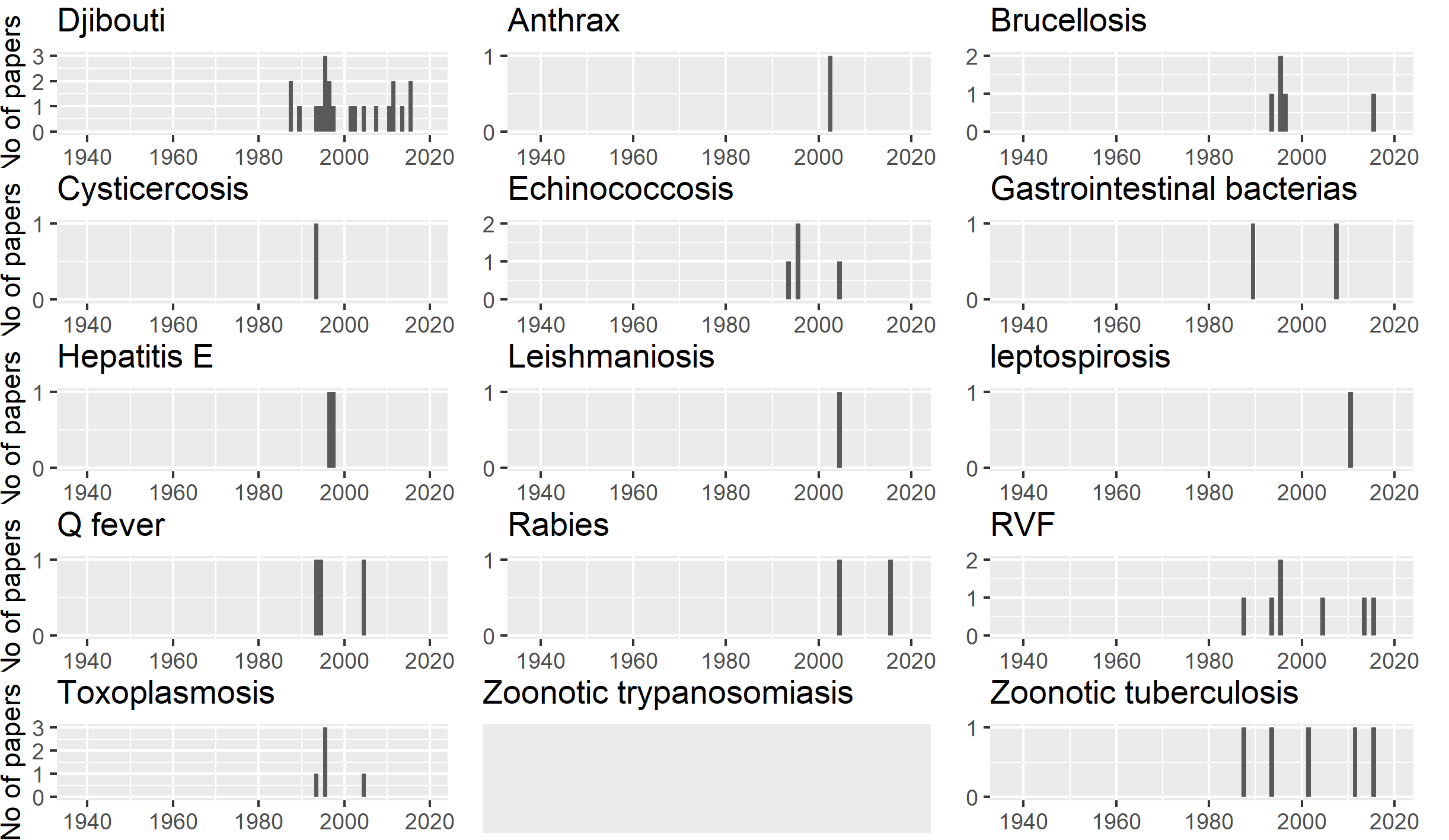


(H)


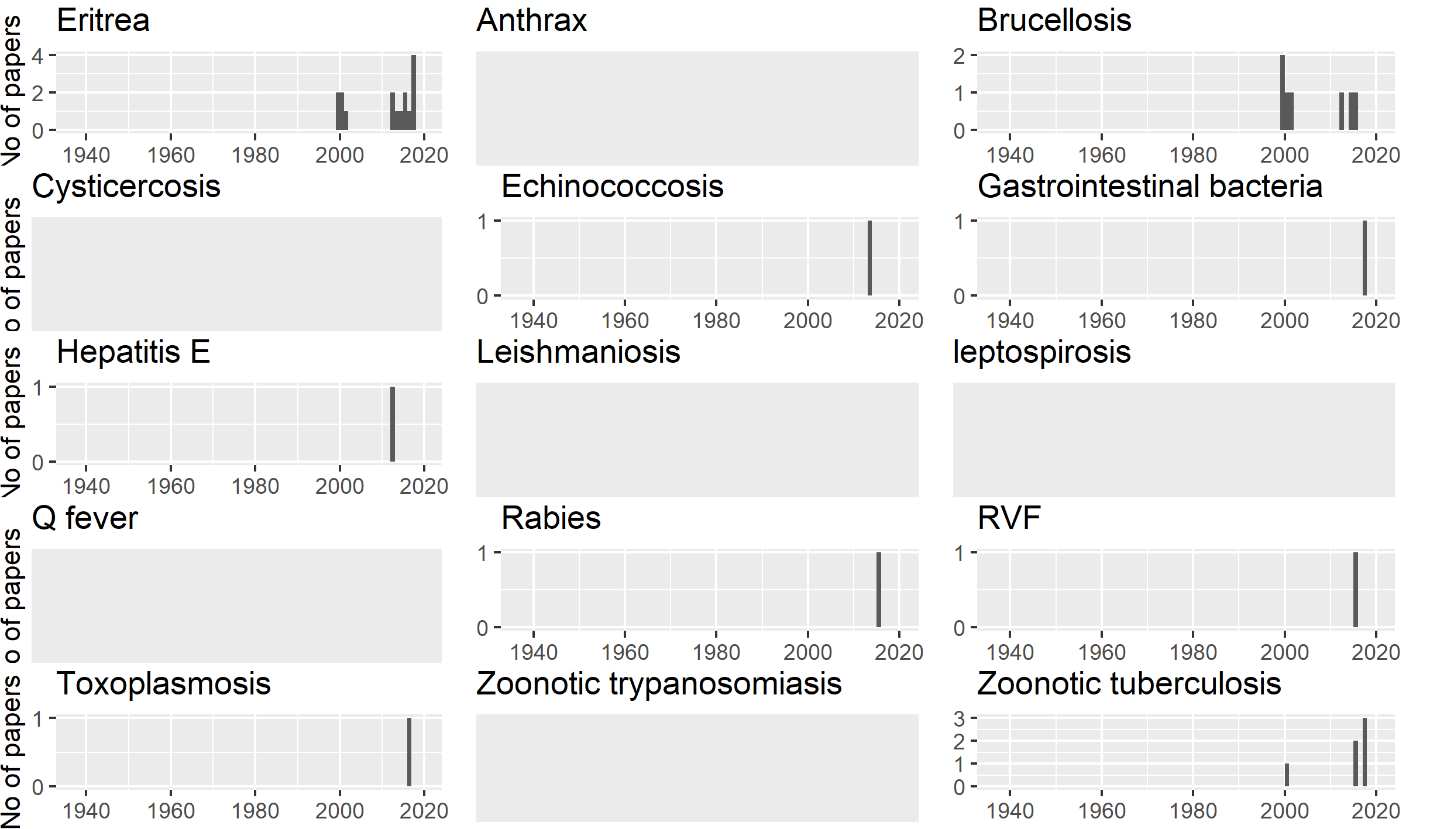


(I)


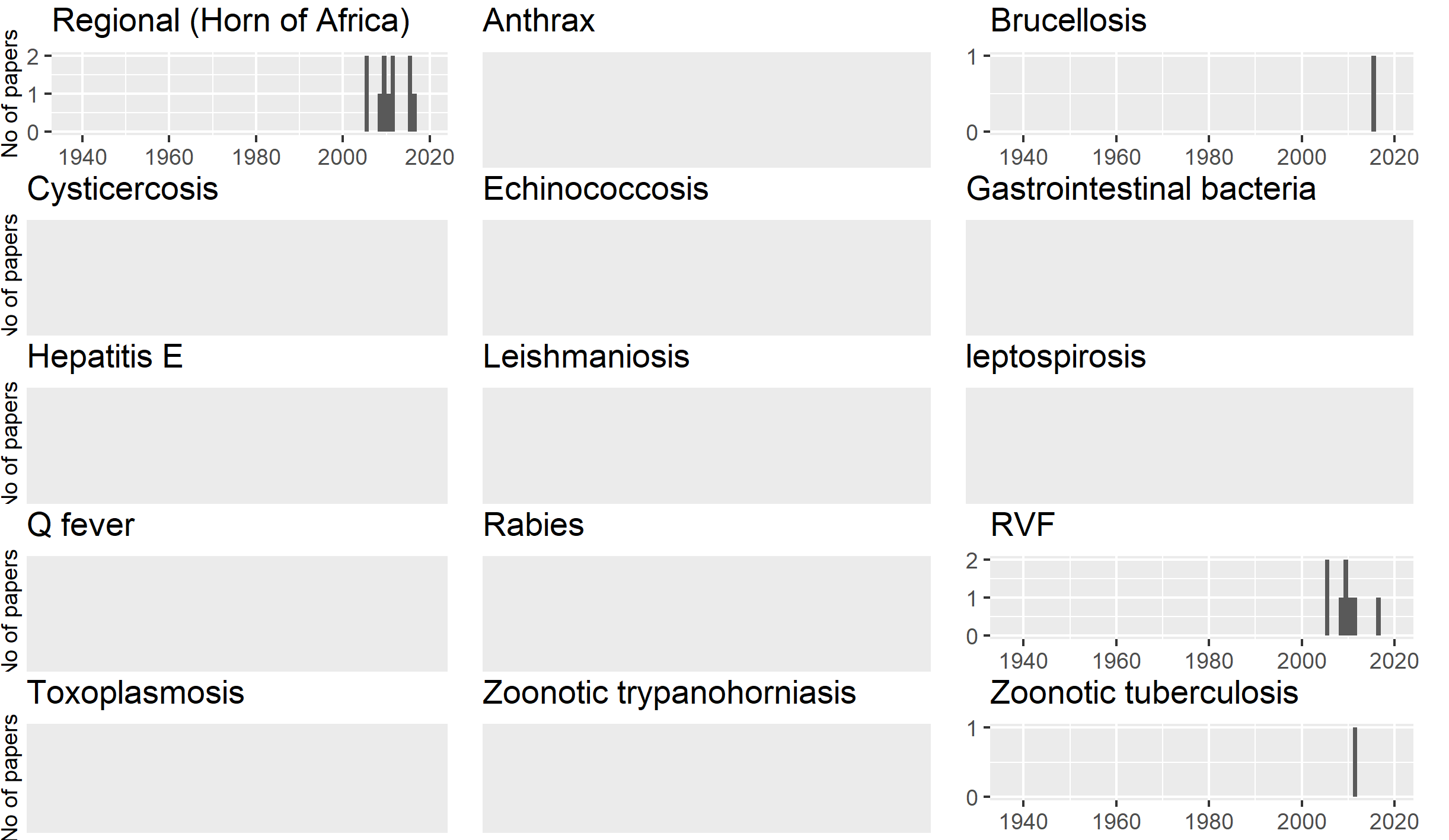

Supplement: S1 Fig — Note: The bars in red represent the year the concerned zoonoses were prioritized in respective national workshops. In cases where no publications were produced that year, the year is indicated by a star. A solid light grey plot means there is no publication for a given disease in this country. (DOCX) [file pntd.0009607.s007.docx]
