# Supplementary material for: One hundred years of zoonoses research in the Horn of Africa: A scoping review
Source: PLoS Negl Trop Dis. 2021 Jul 16;15(7):e0009607. doi: 10.1371/journal.pntd.0009607 (PMC8318308; doi:10.1371/journal.pntd.0009607)
Supplement: Attachment — Submitted filename: Response to reviewers.docx [file pntd.0009607.s009.docx]

Dear Editor and Reviewers,

We thank the Editor and the Reviewers for their constructive and encouraging comments. We hope that our response will be satisfying and that the modifications we have made to the manuscript will make it suitable for publication. Please note that we have taken this review phase as an opportunity to revise some wording across the manuscript with the only objective to improve the writing and the clarity of the text for future readers. No substantial changes were made outside of those described below to answer the reviewers’ queries.

Best wishes,

On behalf of all the authors, Lisa Cavalerie

**Responses to reviewers’ comments**

**Key Review Criteria Required for Acceptance?** As you describe the new analyses required for acceptance, please consider the following:

**Methods**

-Are the objectives of the study clearly articulated with a clear testable hypothesis stated?

-Is the study design appropriate to address the stated objectives?

-Is the population clearly described and appropriate for the hypothesis being tested?

-Is the sample size sufficient to ensure adequate power to address the hypothesis being tested?

-Were correct statistical analysis used to support conclusions?

-Are there concerns about ethical or regulatory requirements being met?

Reviewer #1: Study objectives, study design, and analyses were carried out sufficiently in accordance with PRISMA guidelines. No concerns around ethical or regulatory requirements.

No further action required.

Reviewer #2: The methods are overall clearly stated and their limitations also openly described in the discussion. I have only minor comments:

-Please state if there were any language restrictions made in the search -Please state the R version used

There was no restriction on language in the database search strategy. This is now clarified in line 178-179 of the document with track-changes and all mark-up view, showing all revisions in line. As stated in line 171, at the stage of paper selection for inclusion, only English and French papers were retained. R version 3.5.1 was used (added line 229).

--------------------

**Results**

-Does the analysis presented match the analysis plan?

-Are the results clearly and completely presented?

-Are the figures (Tables, Images) of sufficient quality for clarity?

Reviewer #1: Presented analyses match the outlined analysis plan. Results are clearly presented and support the conclusions being discussed. Figure 4 is difficult to see. The nodes could be more clearly defined, as could the link thickness.

We have updated Figure 4 (now Figure 5) to make it more clear.

Reviewer #2: - The results are rather thorough and matching each research objective.

- I was hoping however, while reading through the results, to see a figure with publications over time. The figures picturing publications overtime are presented in details in the appendix, but it would be nice to also have an overall one in the main manuscript (I would suggest publications over time per country, not necessarily per disease in the main text). On the figures showing number of publications over time, I would add an arrow for when the national list of priority zoonoses was established. That would allow the reader to reflect on your comment on how research on zoonotic diseases does not always fit the list of priority zoonoses established by the government, in light of when one happened compared to the other. Maybe we would notice an increase of publications on a priority disease after official prioritization, maybe not, but the info is missing.

We have added the figure presenting publications over time per country in the main text with red bars to indicate the year the zoonoses prioritisation workshop took place (Fig 2). We had originally decided not to include this given similarities with another paper ([Kemunto *et al.* 2018)](https://www.ncbi.nlm.nih.gov/pmc/articles/PMC6215645/), however we agree with the Reviewer that this provides useful context.

- It is not clear to me why only 1071 papers out of 2055 were included in the authorship analysis. Can the authors clarify in the text?

Given the high number of papers (2055), we needed to automate the authorship analysis. We used an R package to do this, but it only handles papers indexed in PubMed. Hence we focused this analysis on a smaller subset of papers obtained from PubMed (1071), which are representative of the entire set. This explanation is added at lines 223-226.

--------------------

**Conclusions**

-Are the conclusions supported by the data presented?

-Are the limitations of analysis clearly described?

-Do the authors discuss how these data can be helpful to advance our understanding of the topic under study?

-Is public health relevance addressed?

Reviewer #1: The conclusions are supported by data presented. The points I would wish for the authors to expand upon are outlined in the Summary and General Comments below.

See responses below.

Reviewer #2: The discussion and conclusions are relevant and supported by the data presented.

No further action required.

--------------------

**Editorial and Data Presentation Modifications?**

Use this section for editorial suggestions as well as relatively minor modifications of existing data that would enhance clarity. If the only modifications needed are minor and/or editorial, you may wish to recommend “Minor Revision” or “Accept”.

Reviewer #1: See Summary and General Comments.

See responses below.

Reviewer #2: (No Response)

No further action required.

--------------------

**Summary and General Comments**

Use this section to provide overall comments, discuss strengths/weaknesses of the study, novelty, significance, general execution and scholarship. You may also include additional comments for the author, including concerns about dual publication, research ethics, or publication ethics. If requesting major revision, please articulate the new experiments that are needed.

Reviewer #1: In this piece, authors present a comprehensive scoping review of zoonoses research in the Horn of Africa. This is well-done and thorough review, which answers important questions existing about the nature of interdisciplinary collaboration and reporting,, which is highly relevant to the One Health reporting guidelines and One Health frameworks being pushed forward in recent years. I appreciate the authors’ interpretation of their data, and I would only request discussing a few additional points (outlined below).

In addition to empowering local researchers to engage in zoonoses research, I would encourage the discussion to touch on the responsibility of authors from Northern countries to ensure authorship is inclusive of all field members on a team. I suspect contributions from authors in the study area are underrepresented and increasing dialogue within the research community about the importance of acknowledging work from all in-country partners is long overdue; your manuscript provides an opportunity to touch on this important point.

Thank you for this very valuable comment. We have added in the discussion (lines 553-557) a sentence emphasizing the importance of authorship and sample ownership in equitable research collaboration referring to a recently published scoping review by [Faure et al.](https://www.ncbi.nlm.nih.gov/pmc/articles/PMC7796532/#CR55) and the authorship recommendations set by the [ICMJE](http://www.icmje.org/recommendations/archives/).

While it is illustrated in Figure 4, it would be nice if you could verbally expand upon, or share, the list of countries represented in the South-South collaborations. These are indeed more rare than South-North collaborations, and I think these relationships are worth exploring, particularly as it relates to your other points about out-of-country collaboration shaping research landscapes and priorities. Along this same line, when comparing those diseases that are locally prioritized (but not considered global priority), did you see a significant difference in author origin (e.g. local/in-country only vs. involvement of foreign/Global North collaborators)? This relates to your commentary surrounding the involvement of Global North authors shaping the research landscape (line 470), and I fully agree. If it’s within the scope of the review, or perhaps as a follow-up commentary, suggestions and directions for improving representation of local in-country authors would be beneficial. For instance, how underrepresented are in-country others on authorship lists? I couldn’t agree with your statement in lines 486-488 more.

We have added to the supplementary material an excel file (S1 FILE) with the list of all the “links” between countries i.e. the collaborations represented in Figure 5 (ex Figure 4). We have also added in the text, lines 399-401, the list of the top 3 South-South collaborations: Kenya-South Africa, Kenya-Uganda and Kenya-Ethiopia but they only represent 4%, 3% and 3%, respectively of the South-South collaborations which are very scattered.

We agree that there is a need to improve representation of local in-country authors, and we hope we will be able to expand on this topic in a follow-up paper. The authorship analysis presented here is meant to be a first exploration of the North-South dynamics behind zoonoses research in the Horn of Africa. We agree that this section could be complemented in a follow-up work with further analysis on the location of first and last authors but also more details by disease.

Please describe inclusion/exclusion criteria for biotic environmental factors more specifically. This is particularly important owing to the number of vector-borne diseases covered in this scoping review. Please break down the 68% of biotic papers reporting on free-living arthropod vectors to investigate which arthropods were most reported on. I’m a bit surprised there aren’t more papers listed here, considering the burden of vector-borne disease in this region. It’s worth teasing this apart a bit, if the editor feels it is within the scope of this paper.

The definition of biotic/abiotic environment sources is provided in S3 Table:

- biotic source (e.g. free-living arthropods, plants, plant-based food, manure)
- abiotic source (e.g. water/soil/surfaces)

When studies were only reporting on ectoparasites found on animals, e.g. on-host ticks, fleas and lice (n=43 papers), we considered this was not reflective of the environment but rather of the animal domain. All papers reporting data on free-living arthropods (n=143) were classified as reporting data on biotic sources (n=211) and then aggregated as environment (n=285 papers). We have not extracted the arthropod species reported on, and this would require systematically reading individual papers, which was not part of the research design. However, by looking at the disease these papers are reporting on we can have some idea of the arthropods involved: 60 of these 143 papers are on RVF vectors (Aedes spp, Culex spp, etc.) and 34 on trypanosomiasis vectors (essentially Glossina spp). This information has been added to the main text (lines 322-324)

Lines 428-432: Could you expand upon how VBD having higher degree of tri-sector reporting indicates the COHERE checklist is too restrictive? This is an interesting point and worth going into a bit more detail on, since not all readers may be familiar with the specifics of the COHERE checklist.

In our scoping review we have identified that only 73 papers out of 2055 report on all three One Health domains. Zoonotic trypanosomiasis and RVF are the focus of 21 and 14 of them respectively. Studying diseases which cycle strongly depend on the environment like vector-borne diseases makes them more amenable to classification as ‘One Health’ research according to COHERE. Meanwhile, papers reporting on a disease like rabies will not achieve the ‘One Health’ criteria suggested by COHERE as these have limited opportunity to incorporate the environment domain. This seems somewhat counterproductive when One Health zoonotic disease prioritisation exercises by stakeholders never fail to prioritise rabies. We have revised the wording to make this clearer (see modification at lines 475-494).

The authors discuss the importance of reporting on all three sectors/domains (humans, animals and the environment), which certainly would help frame the results of studies through a more holistic lens and help check off the COHERE guidelines. However, reporting on all three domains often requires additional resources or additional investigation and analyses that may be cost and time-prohibitive. I wonder if there would be a way to anecdotally describe patterns you appreciated in those papers that DID report on all three domains, citing and referencing these papers specifically as a means of encouraging other authors to pursue similar frameworks. This may be a more tangible and inspirational example than pointing authors aspiring to report through a One Health framework to the COHERE guidelines.

This idea has merit, but to be able to reply precisely and exhaustively, it would require systematically reading the full papers, which was not part of the research design. In order to accommodate this comment, we have added some examples of papers reporting on all 3 domains (line 432-449).

In light of these comments, we have somewhat revisited the discussion and made some formatting revisions including moving some paragraphs around for the sake of clarity.

Reviewer #2: I do not have further comments. The manuscript is very well written, the authors are transparent about their methods and results, and these results are needed to highlight solutions for a way forward in One Health research especially in the global South.

No further action required.

--------------------

PLOS authors have the option to publish the peer review history of their article ([what does this mean?](https://journals.plos.org/plosntds/s/editorial-and-peer-review-process#loc-peer-review-history)). If published, this will include your full peer review and any attached files.

If you choose “no”, your identity will remain anonymous but your review may still be made public.

**Do you want your identity to be public for this peer review?** For information about this choice, including consent withdrawal, please see our [Privacy Policy](https://www.plos.org/privacy-policy).

Reviewer #1: Yes: Anna Fagre

Reviewer #2: No

Figure Files:

While revising your submission, please upload your figure files to the Preflight Analysis and Conversion Engine (PACE) digital diagnostic tool, <https://pacev2.apexcovantage.com>. PACE helps ensure that figures meet PLOS requirements. To use PACE, you must first register as a user. Then, login and navigate to the UPLOAD tab, where you will find detailed instructions on how to use the tool. If you encounter any issues or have any questions when using PACE, please email us at figures@plos.org.

Data Requirements:

Please note that, as a condition of publication, PLOS' data policy requires that you make available all data used to draw the conclusions outlined in your manuscript. Data must be deposited in an appropriate repository, included within the body of the manuscript, or uploaded as supporting information. This includes all numerical values that were used to generate graphs, histograms etc.. For an example see here: <http://www.plosbiology.org/article/info%3Adoi%2F10.1371%2Fjournal.pbio.1001908#s5>.

Reproducibility:

To enhance the reproducibility of your results, we recommend that you deposit your laboratory protocols in protocols.io, where a protocol can be assigned its own identifier (DOI) such that it can be cited independently in the future. Additionally, PLOS ONE offers an option to publish peer-reviewed clinical study protocols. Read more information on sharing protocols at <https://plos.org/protocols?utm_medium=editorial-email&utm_source=authorletters&utm_campaign=protocols>

References

Please review your reference list to ensure that it is complete and correct. If you have cited papers that have been retracted, please include the rationale for doing so in the manuscript text, or remove these references and replace them with relevant current references. Any changes to the reference list should be mentioned in the rebuttal letter that accompanies your revised manuscript. If you need to cite a retracted article, indicate the article's retracted status in the References list and also include a citation and full reference for the retraction notice.

__________________________________________________

In compliance with data protection regulations, you may request that we remove your personal registration details at any time. (Use the following URL: <https://www.editorialmanager.com/pntd/login.asp?a=r>). Please contact the publication office if you have any questions.
